# Capsular Polysaccharide Is Essential for the Virulence of the Antimicrobial-Resistant Pathogen Enterobacter hormaechei

**DOI:** 10.1128/mbio.02590-22

**Published:** 2023-02-13

**Authors:** Amelia St. John, Andrew I. Perault, Sabrina I. Giacometti, Alexis G. Sommerfield, Ashley L. DuMont, Keenan A. Lacey, Xuhui Zheng, Julia Sproch, Chris Petzold, Kristen Dancel-Manning, Sandra Gonzalez, Medini Annavajhala, Colleen Beckford, Nathalie Zeitouni, Feng-Xia Liang, Harm van Bakel, Bo Shopsin, Anne-Catrin Uhlemann, Alejandro Pironti, Victor J. Torres

**Affiliations:** a Department of Microbiology, New York University Grossman School of Medicine, New York, New York, USA; b Antimicrobial-Resistant Pathogens Program, New York University Grossman School of Medicine, New York, New York, USA; c Department of Cell Biology, New York University Grossman School of Medicine, New York, New York, USA; d Microscopy Laboratory, Division of Advanced Research Technologies, New York University Langone Health, New York, New York, USA; e Department of Medicine, Division of Infectious Diseases, Columbia University Medical Center, New York, New York, USA; f Department of Genetics and Genomic Sciences, Icahn School of Medicine at Mount Sinai, New York, New York, USA; g Icahn Genomics Institute, Icahn School of Medicine at Mount Sinai, New York, New York, USA; h Department of Medicine, Division of Infectious Diseases, New York University Grossman School of Medicine, New York, New York, USA; i Microbial Computational Genomic Core Lab, Department of Microbiology, New York University Grossman School of Medicine, New York, New York, USA; University of Georgia

**Keywords:** Enterobacter, serum, complement, capsule, antimicrobial resistance, pathogenesis, serum resistance

## Abstract

Nosocomial infections caused by multidrug-resistant (MDR) Enterobacter cloacae complex (ECC) pathogens are on the rise. However, the virulence strategies employed by these pathogens remain elusive. Here, we study the interaction of ECC clinical isolates with human serum to define how this pathogen evades the antimicrobial action of complement, one of the first lines of host-mediated immune defense. We identified a small number of serum-sensitive strains, including Enterobacter hormaechei strain NR3055, which we exploited for the *in vitro* selection of serum-resistant clones. Comparative genomics between the serum-sensitive NR3055 strain and the isolated serum-resistant clones revealed a premature stop codon in the *wzy* gene of the capsular polysaccharide biosynthesis locus of NR3055. The complementation of *wzy* conferred serum resistance to NR3055, prevented the deposition of complement proteins on the bacterial surface, inhibited phagocytosis by human neutrophils, and rendered the bacteria virulent in a mouse model of peritonitis. Mice exposed to a nonlethal dose of encapsulated NR3055 were protected from subsequent lethal infections by encapsulated NR3055, whereas mice that were previously exposed to unencapsulated NR3055 succumbed to infection. Thus, capsule is a key immune evasion determinant for *E. hormaechei*, and it is a potential target for prophylactics and therapeutics to combat these increasingly MDR human pathogens.

## INTRODUCTION

Within the family Enterobacteriaceae, the genus Enterobacter contains emerging bacterial pathogens of clinical concern (1). Enterobacter species are Gram-negative bacteria that are commonly found in soil (2), water (3), sewage (4), as well as in association with plants (5), and they are residents of the normal human enteric microbiota (6, 7). The prevalence and importance of Enterobacter spp. as nosocomial pathogens has increased, especially in immunocompromised individuals (6, 8). Notably, neonates and preterm infants, as well as patients in intensive care units and postsurgical patients, are most often affected by nosocomial Enterobacter infections (6, 9, 10). Enterobacter spp. are included in a group of “priority status” organisms that are known as ESKAPE pathogens (Enterococcus faecium, Staphylococcus aureus, Klebsiella pneumoniae, Acinetobacter baumannii, Pseudomonas aeruginosa, and Enterobacter spp.) (11–13). These bacteria are threats to human health due to their acquired resistance mechanisms against a multitude of antibiotics, including last-resort interventions (11).

Enterobacter spp., specifically the Enterobacter cloacae complex (ECC), have acquired mechanisms of carbapenem and other β-lactam resistance (11, 12, 14). The ECC is composed of at least seven species, of which Enterobacter cloacae and Enterobacter hormaechei are the most clinically relevant ECC human pathogens (1, 6, 8, 14). These species can cause a wide range of infections, including, but not limited to, bloodstream, intraabdominal, urinary tract, gastrointestinal, and lung infections, all with the potential of leading to bacteremia with case fatality rates as high as 40% (6, 14–16). Many of the disease-causing *E. hormaechei* strains belong to the clonal groups of carbapenem-resistant ECC (CREC) and extended-spectrum β-lactamase (ESBL)-producing ECC sequence types (ST) 78 and ST171 (14, 17). These clonal groups are associated with a rapid increase in MDR Enterobacter spp. infections, and they have heightened the need for understanding Enterobacter virulence as well as the underlying host response in order to design new and effective treatments and interventions for the infections they cause. Despite the increasing concern over Enterobacter infections, the molecular mechanisms promoting Enterobacter pathogenesis are largely unknown.

Serum, the fluid component of blood, harbors complement proteins that are involved in the complement cascade, a first line of defense against microbial pathogens in humans (18, 19). Complement is involved in the defense against Gram-negative pathogens via the formation of a lytic pore in the bacterial cell membrane that is known as the membrane attack complex (MAC) (18, 20, 21). Complement cascade initiation occurs through the activation of three pathways: the classical pathway (CP), the lectin pathway (LP), and the alternative pathway (AP) (18, 22, 23). The deposition of complement protein 3b (C3b) on the bacterial cell surface to drive the production of C5 convertases is a necessary early step in the complement cascade, and it is where the CP, LP, and AP converge (21, 24). The C5 convertases drive downstream cleavage events of complement proteins, thereby resulting in MAC formation (18). Although serum-mediated killing through complement cascade activation is a primary immune defense against bacterial pathogens, many bacteria possess complement evasion strategies (18, 20). Among these strategies is the production of capsular polysaccharide (CPS) (18, 20). CPS interferes with the initiation of the complement cascade by limiting the availability of surface antigens to serum factors for complement binding (18). Clinical isolates of Escherichia coli and K. pneumoniae commonly produce CPS, and their mechanisms of resistance to serum and complement-mediated killing have been studied (20, 21, 25). However, the mechanisms of complement evasion by Enterobacter spp. are unknown.

To investigate if and how Enterobacter resists serum-mediated killing, we screened 96 recently collected clinical isolates for their abilities to withstand human complement antibacterial activity. Although most of these isolates exhibited notable serum resistance, a small number of isolates were highly sensitive to complement killing. One ST78 isolate, NR3055, was identified as highly serum sensitive, and it was used to identify mechanisms of complement resistance. Through selecting for serum-resistant (SR) clones under the pressure of human serum and conducting comparative genomics between NR3055 and selected NR3055 SR clones, we identified a premature stop codon in the *wzy* gene of the *cps* capsular polysaccharide biosynthesis locus of NR3055. While NR3055 was avirulent in human whole blood and *in vivo* infection models, complementation of the *wzy* gene conferred serum resistance and virulence to NR3055. Moreover, we show that recurrent infections with *E. hormaechei* induced immune memory, which protects against a lethal dose of encapsulated NR3055 in a CPS-dependent manner. In summary, we identify CPS as a critical virulence factor for promoting the pathogenesis of *E. hormaechei*, and we show that targeting CPS may be a prophylactic strategy by which to prevent Enterobacter infections.

## RESULTS

### Clinical isolates of Enterobacter spp. display diverse susceptibility to human serum bactericidal activity.

**FIG 1:**
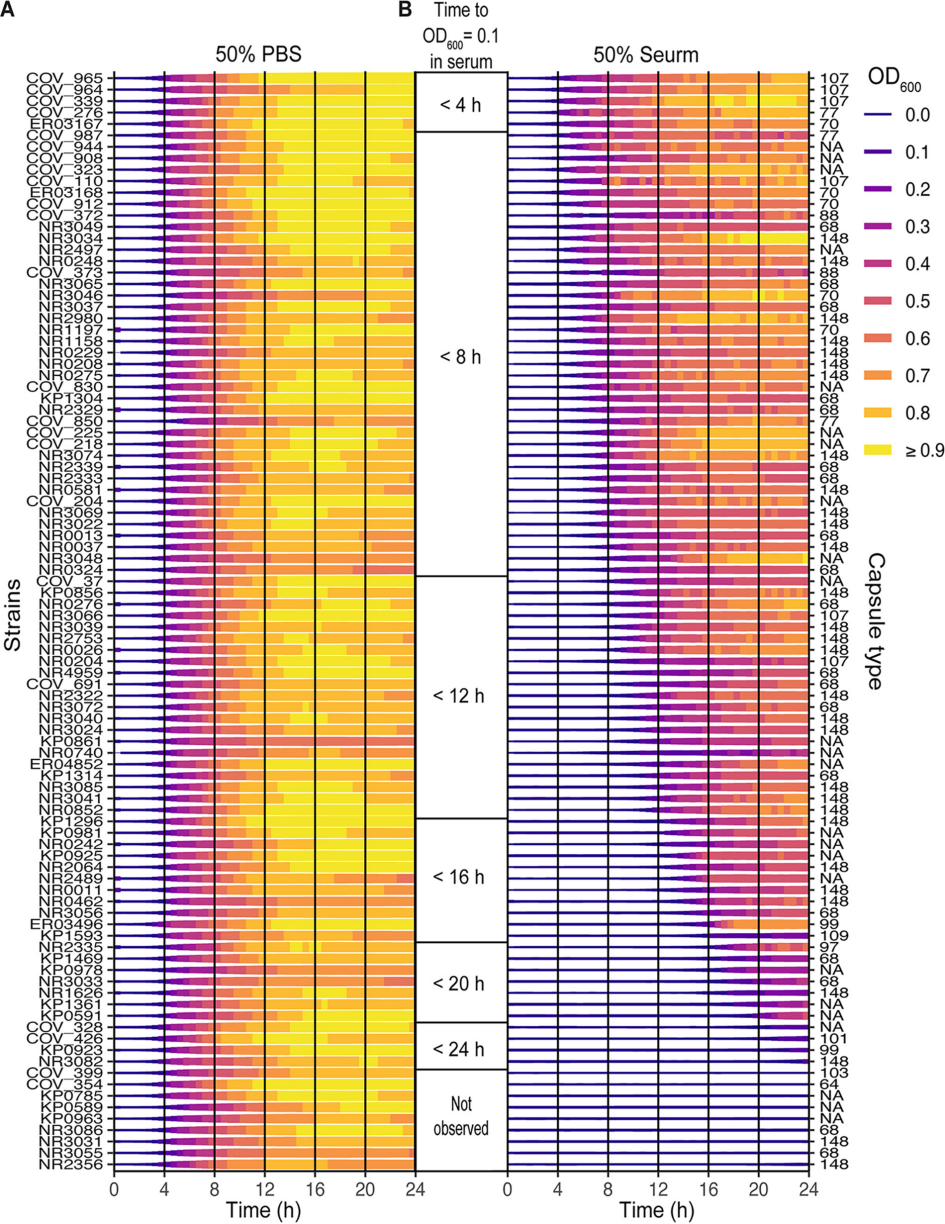
Serum susceptibility of Enterobacter spp. clinical isolates. 96 Enterobacter spp. clinical isolates were cultured in either 50% TSB + 50% PBS (A) or 50% TSB + 50% commercial human serum (B). The OD_600_ of each culture was measured every 30 min for 24 h. Isolates are sorted according to the time it took the cultures to reach an OD_600_ of 0.1 in serum and are grouped according to the 4 h time frame in which this optical density was reached by the culture (B). The most serum-resistant isolates appear at the top of the figure, whereas the most sensitive isolates are at the bottom. Readings of the OD_600_ value over time are mapped to the color and thickness of the horizontal line segments representing the individual strains. The thin, purple line segments represent low OD_600_ values, whereas the thick, yellow line segments represent high OD_600_ values. Each strain is represented by two biological replicates that were averaged for this figure. The strain names are listed to the left, and the capsule types are listed to the right. NA, capsule type could not be determined. The figure was plotted using R v. 4.1.1 and ggplot v. 3.3.5.

Little is known about the pathogenesis of Enterobacter spp. To investigate Enterobacter serum resistance from a population standpoint, we screened a previously published collection of Enterobacter spp. clinical isolates (14), supplemented with newly collected Enterobacter spp. clinical isolates (a total of 96 isolates), for their abilities to grow in tryptic soy broth (TSB) and in human serum. For the most part, all of the strains had similar growth characteristics when grown in TSB with 50% phosphate-buffered saline (PBS) (Fig. 1A). In contrast, when isolates were grown in TSB with 50% commercial pooled normal human serum (commercial serum), there was an extensive diversity in serum susceptibility (Fig. 1B). Most isolates were highly serum-resistant, exhibiting uninhibited growth, whereas other isolates were highly serum sensitive and unable to grow (Fig. 1B).

**FIG 2:**
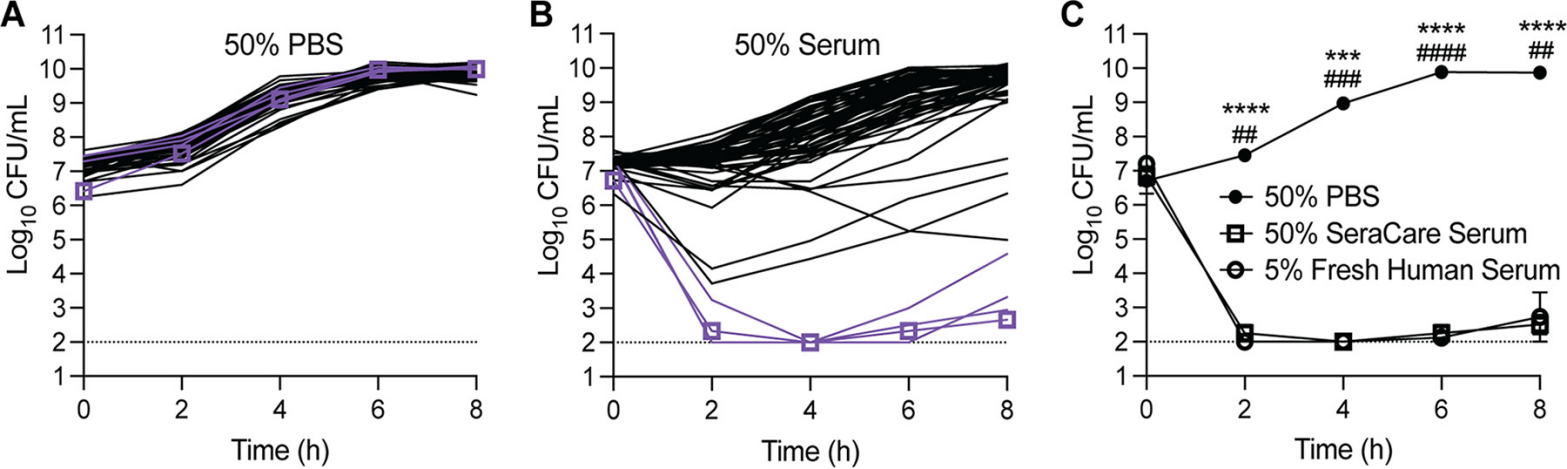
Serum bactericidal activity against 62 Enterobacter spp. clinical isolates (A and B) Enterobacter clinical isolates were cultured in either 50% TSB + 50% PBS (A) or 50% TSB + 50% commercial serum (B). The cultures were serially diluted in PBS and plated on TSA for CFU counts every 2 h for 8 h. Four highly serum-sensitive strains are shown in purple. The serum-sensitive *E. hormaechei* strain NR3055 is shown in purple, with purple squares at each time point. Each strain is represented by two biological replicates. The limit of detection for this assay is 100 CFU/mL (dotted lines). (C) NR3055 was cultured in 50% TSB + 50% PBS, 50% TSB + 50% pooled human serum, or 50% TSB + 45% PBS + 5% fresh pooled human serum. The cultures were serially diluted in PBS and plated on TSA for CFU counts every 2 h for 8 h. Each growth condition is represented by three biological replicates. The limit of detection for this assay is 100 CFU/mL (dotted lines). The error bars indicate the standard error of the mean (SEM). Statistical significance was determined via a two-way ANOVA with Dunnett’s multiple-comparison test. Asterisks represent 50% TSB + 50% PBS compared to 50% TSB + 50% pooled human serum, and pound signs represent 50% TSB + 50% PBS compared to 50% TSB + 45% PBS + 5% fresh human serum (****/####, *P ≤ *0.0001; ***/###, *P ≤ *0.001; **/##, *P ≤ *0.01).

We chose 62 isolates representing the diversity of serum susceptibility shown in the initial screen (Fig. 1B) for further examination. These 62 isolates were cultured in TSB with 50% PBS (Fig. 2A) or 50% commercial serum (Fig. 2B), and serum-mediated bacterial killing was measured by plating for viable bacteria. Like the growth-curve screen, the isolates displayed a large diversity in serum susceptibility, though this assay enabled us to quantify the number of bacteria being killed by the serum, rather than the growth inhibition (Fig. 2A and B). Four of the 62 isolates were identified as highly serum sensitive (Fig. 2B, highlighted in purple).

Among the serum-sensitive isolates, we selected strain NR3055 for further study (Fig. 1 and 2). The bacterial burden of NR3055 was reduced by five logs within the first 2 h of culture with 50% commercial human serum (Fig. 2C). Of note, similar results were obtained when culturing NR3055 in 5% freshly isolated human serum, a serum that was minimally processed and exhibited higher complement activity than the commercial serum (Fig. 2C).

### *In vitro*-selected NR3055 clones are highly resistant to killing by human serum.

**FIG 3:**
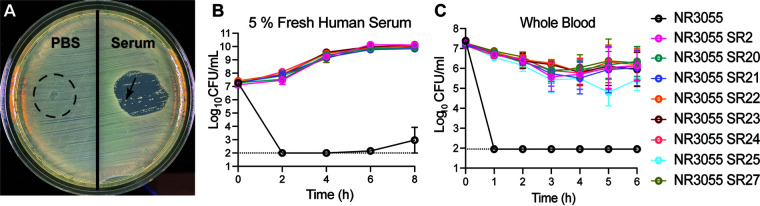
*In vitro* selection of NR3055 serum-resistant clones. (A) PBS and 100% commercial pooled human serum were spotted on a TSA plate on which NR3055 cells were spread. Bacteria grew uninhibited where PBS was spotted, whereas the spotted serum created a zone of clearance. Colonies that arose within the zone of clearance were selected and screened a second time for serum resistance, and those that grew uninhibited were labeled as serum-resistant (SR) clones. The arrow indicates a selected, possibly serum-resistant clone. (B) The eight *in vitro*-selected NR3055 SR clones and NR3055 were cultured in 50% TSB + 45% PBS + 5% fresh pooled human serum. The cultures were serially diluted in PBS and plated on TSA for CFU counts every 2 h for 8 h. Each clone is represented by three biological replicates. The limit of detection for this assay is 100 CFU/mL (dotted line). The error bars indicate the SEM. (C) The eight *in vitro*-selected NR3055 SR clones and NR3055 were used to infect whole human blood. The cultures were serially diluted in PBS and plated on TSA for CFU counts every hour for 6 h. Each clone is represented by two biological replicates with two blood donors per replicate. The limit of detection for this assay is 90 CFU/mL (dotted line). The error bars indicate the SEM.

To understand the serum-resistant phenotype, we isolated serum-resistant clones of NR3055 *in vitro*. NR3055 cells were spread onto tryptic soy agar (TSA), and 100% commercial human serum was spotted on the agar. After overnight incubation, the serum yielded a zone of clearance in which the vast majority of NR3055 cells had been killed, yet some colonies grew within that zone (Fig. 3A). We screened 348 of these colonies for serum resistance and, while most remained serum sensitive, eight serum-resistant (SR) clones were identified and named SR2, SR20, SR21, SR22, SR23, SR24, SR25, and SR27. Importantly, these eight SR clones originated from multiple NR3055 parental colonies and their broth cultures. The *in vitro*-selected NR3055 SR clones were highly serum-resistant, and they displayed uninhibited growth in fresh human serum (Fig. 3B).

Next, the NR3055 SR clones were tested for their ability to withstand killing in an *ex vivo* bacteremia model using freshly isolated human blood (26). Blood was collected in anticoagulant coated tubes, and it was infected with NR3055 and the eight SR clones. The bacterial burden was measured over time. The NR3055 SR clones were resistant to whole-blood killing, whereas the parental NR3055 strain was killed to the limit of detection within 1 h of culture (Fig. 3C).

### NR3055 SR2 produces a capsule-like surface structure.

**FIG 4:**
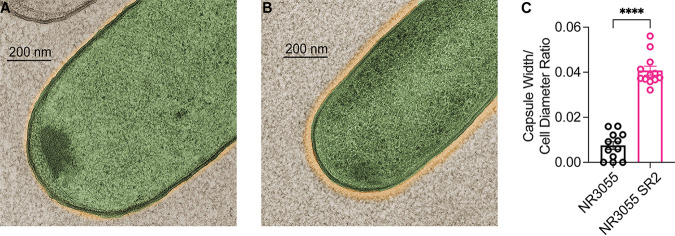
Transmission electron microscopy identified a capsule-like structure surrounding NR3055 SR2. TEM images of NR3055, which exhibited minimal production of the capsule-like structure (A), and NR3055 SR2, which exhibited a thicker zone of putative CPS surrounding the cell (B). The images are pseudocolored based on pixel density and contrast, with yellow indicating the putative capsule and green indicating the cell body. The images are representative of at least 12 cells per strain. Additional images can be found in Fig. S2. (C) Quantification of the width of the capsule-like structures, relative to the diameter of the cell bodies, of 12 NR3055 cell images and 12 NR3055 SR2 cell images. The error bars indicate the SEM. Statistical significance was determined via a Mann-Whitney nonparametric *t* test (****, *P ≤ *0.0001).

To understand the mechanism of SR2-mediated serum resistance, parental NR3055 and NR3055 SR2 were imaged using transmission electron microscopy (TEM), following high-pressure freeze substitution. High-resolution images revealed a putative CPS-like structure surrounding the surfaces of the NR3055 SR2 cells, a structure that was mostly absent on the NR3055 cells (Fig. 4A and B). The capsule-width to cell-diameter ratio was approximately five times larger on NR3055 SR2, compared to NR3055 (Fig. 4C). Given the location of the shell, we hypothesized that NR3055 SR2 produces a protective polysaccharide capsule-like structure that is lacking in the parental strain.

10.1128/mbio.02590-22.2 FIG S2 TEM images. Representative TEM images of two NR3055 cells (A and B) and two NR3055 SR2 cells (C and D), both taken at 57,000× magnification. Cyan lines span the cell diameters, and magenta lines (four per cell) span the zones of putative capsule. A quantification of the ratio of the capsule width to the cell diameter is shown in Fig. 4C. Download FIG S2, PDF file, 0.5 MB. Copyright © 2023 St. John et al. 2023 St. John et al. https://creativecommons.org/licenses/by/4.0/ This content is distributed under the terms of the Creative Commons Attribution 4.0 International license.

### The NR3055 genome contains a premature stop codon in the *wzy* gene of the *cps* capsular biosynthesis gene cluster.

To define the genetic determinants of serum resistance within our collection, especially among NR3055 and its serum-resistant clones, we sequenced the genomes of all 96 of the isolates shown in our initial screen as well as those of the 8 NR3055 SR clones using Illumina short-read technology. Additionally, we sequenced NR3055 and NR3055 SR2 using Oxford Nanopore long-read technology. Hybrid short-and long-read assemblies were generated for these two isolates, and short-read assemblies were generated for the remaining strains. We annotated the assemblies computationally with genes, taxonomic lineage, multilocus sequence type (MLST, also ST), and capsule type (Table S1). NR3055 was classified as ST78 *E. hormaechei* with an Enterobacter-NL68 capsule type.

10.1128/mbio.02590-22.4 TABLE S1 Overview of isolates screened and sequenced in this study. The isolates investigated in this study are tabulated. “Site” refers to the body site from which the bacteria were isolated. “Serum resistance group” refers to the grouping of isolates by the times they required to reach an OD_600_ of 0.1 when screened in human serum (Fig. 1). The species are annotated as output by GTDB-Tk (Materials and Methods). MLST was obtained with the PubMLST scheme for Enterobacter cloacae (Materials and Methods). The capsule types were assigned as described in Materials and Methods. Download Table S1, XLSX file, 0.01 MB. Copyright © 2023 St. John et al. 2023 St. John et al. https://creativecommons.org/licenses/by/4.0/ This content is distributed under the terms of the Creative Commons Attribution 4.0 International license.

To better understand the interplay between genomic variants, lineages, capsule types, and serum resistance, we computed a phylogeny for the strains (Fig. S1). We compared the hybrid short- and long-read assembly of NR3055 to its eight SR clones and consistently found only one single nucleotide variant that was present in all of the SR clones, relative to the NR3055 parental genome, with no further nucleotide changes. This nucleotide change repaired a premature stop codon in *wzy* (c.629C>A; p.Ser210*). Wzy is a capsular oligosaccharide polymerase that is encoded by the group 1 capsule biosynthesis locus *cps*, which is found across Enterobacterales pathogens and is well appreciated for its impact on the virulence and serum resistance of Escherichia coli, K. pneumoniae, and Salmonella enterica (27–30). Thus, the identification of a mutation in *wzy* was in line with the observed change in the surface envelope in the parental NR3055 strain (Fig. 4A–C).

10.1128/mbio.02590-22.1 FIG S1 Phylogeny of the isolates screened in this study. A maximum-likelihood phylogeny was computed with the core genome alignment of the isolates screened in this study (Materials and Methods). The phylogeny is midpoint rooted. The tree scale relates the branch length to the number of substitutions per alignment site. The colored ranges indicate (i) serum resistance, as categorized by the times the isolates required to reach an OD_600_ of 0.1 when screened in human serum (Fig. 1), (ii) capsule type (Materials and Methods), (iii) GTDB-Tk species designation (Materials and Methods), and (iv) body site from which the bacteria were isolated. The serum resistance group and isolation site are not available for the NR3055 serum-resistant clones. Download FIG S1, PDF file, 0.2 MB. Copyright © 2023 St. John et al. 2023 St. John et al. https://creativecommons.org/licenses/by/4.0/ This content is distributed under the terms of the Creative Commons Attribution 4.0 International license.

**FIG 5:**
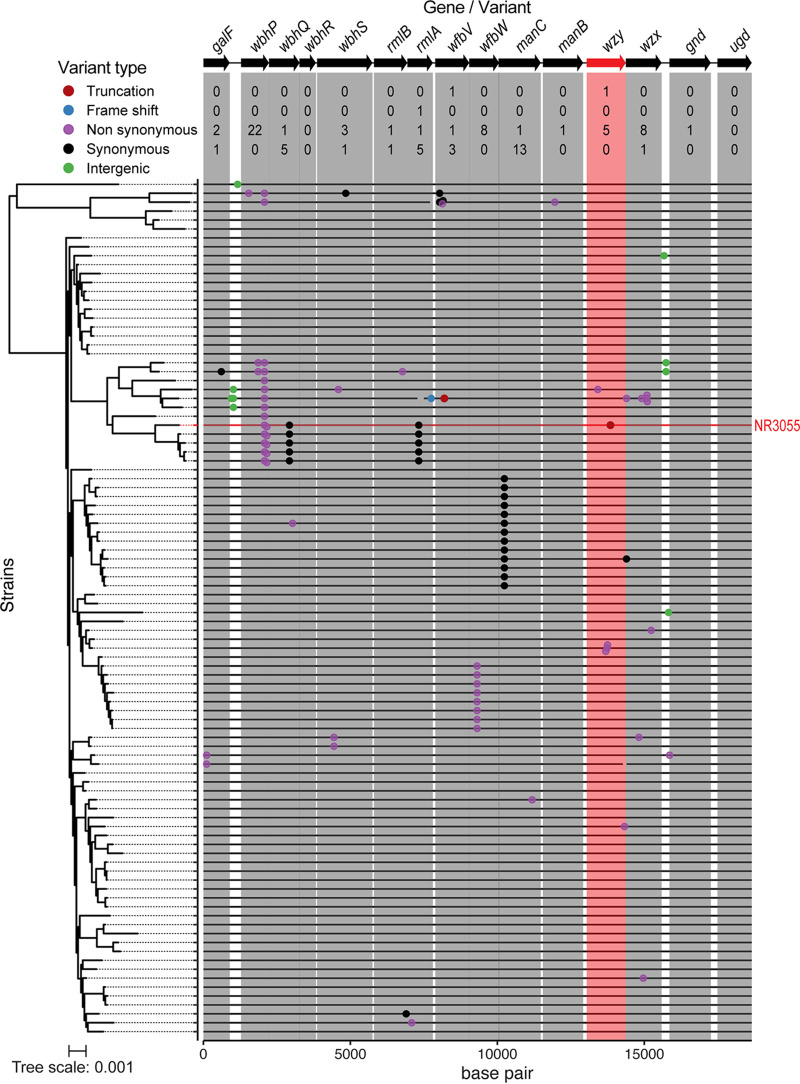
The *cps* locus variants in ST78 *E. hormaechei* isolates. Phylogeny of 97 ST78 *E. hormaechei* assemblies (left). 79 of the phylogenies were obtained from NCBI RefSeq, and 18 stem from the present study. All 97 isolates carry a *cps* locus of type Enterobacter-NL68, which is represented by the gene map on the top. The genetic variants and their locations within the *cps* locus of these assemblies are represented by dots in the middle panel. Variant types are indicated by different colors. Gene boundaries within the central panel are marked by boxes that span the gene length. Below the gene map, the number of variants found in each gene is tabulated by type. The tree scale relates branch length to the number of substitutions per alignment site. The figure was plotted using R v. 4.1.1 and ggplot v. 3.3.5.

To investigate the variability of the *cps* locus among *E. hormaechei*, especially within the lineage and capsule type of NR3055, we downloaded 1,886 publicly available *E. hormaechei* assemblies, of which 979 passed our internal quality control. Among these, we observed a strong association between MLST and capsule type; in 970 of 979 sequence- and capsule-typed assemblies, a given ST translated to one capsule type (although some capsule types were found in various STs) (Table S2). We found 151 assemblies with capsule type Enterobacter-NL68, and these mostly fell within the ST78 lineage (94 assemblies), followed by the ST145 lineage (35 assemblies). Next, we turned to the ST78 lineage and identified the mutations occurring in the *cps* locus (Fig. 5). Non-synonymous mutations were the most prevalent (*n *= 55), and most of them occurred in *wbhP* (*n *= 22), which is a NAD-dependent epimerase/dehydratase that is involved in O-antigen biosynthesis (31). Of note, putative loss-of-function mutations were rarely detected in the *cps* loci (Fig. 5), highlighting the importance of CPS production to the pathogenic potential of *E. hormaechei*.

10.1128/mbio.02590-22.5 TABLE S2 Overview of Enterobacter hormaechei A assemblies obtained from NCBI RefSeq. Enterobacter hormaechei assemblies that were downloaded from NCBI RefSeq are tabulated. MLST was obtained with the PubMLST scheme for Enterobacter cloacae (Materials and Methods). The capsule types were assigned as described in Materials and Methods. Download Table S2, XLSX file, 0.1 MB. Copyright © 2023 St. John et al. 2023 St. John et al. https://creativecommons.org/licenses/by/4.0/ This content is distributed under the terms of the Creative Commons Attribution 4.0 International license.

### Expression of full-length *wzy* confers serum resistance to NR3055.

**FIG 6:**
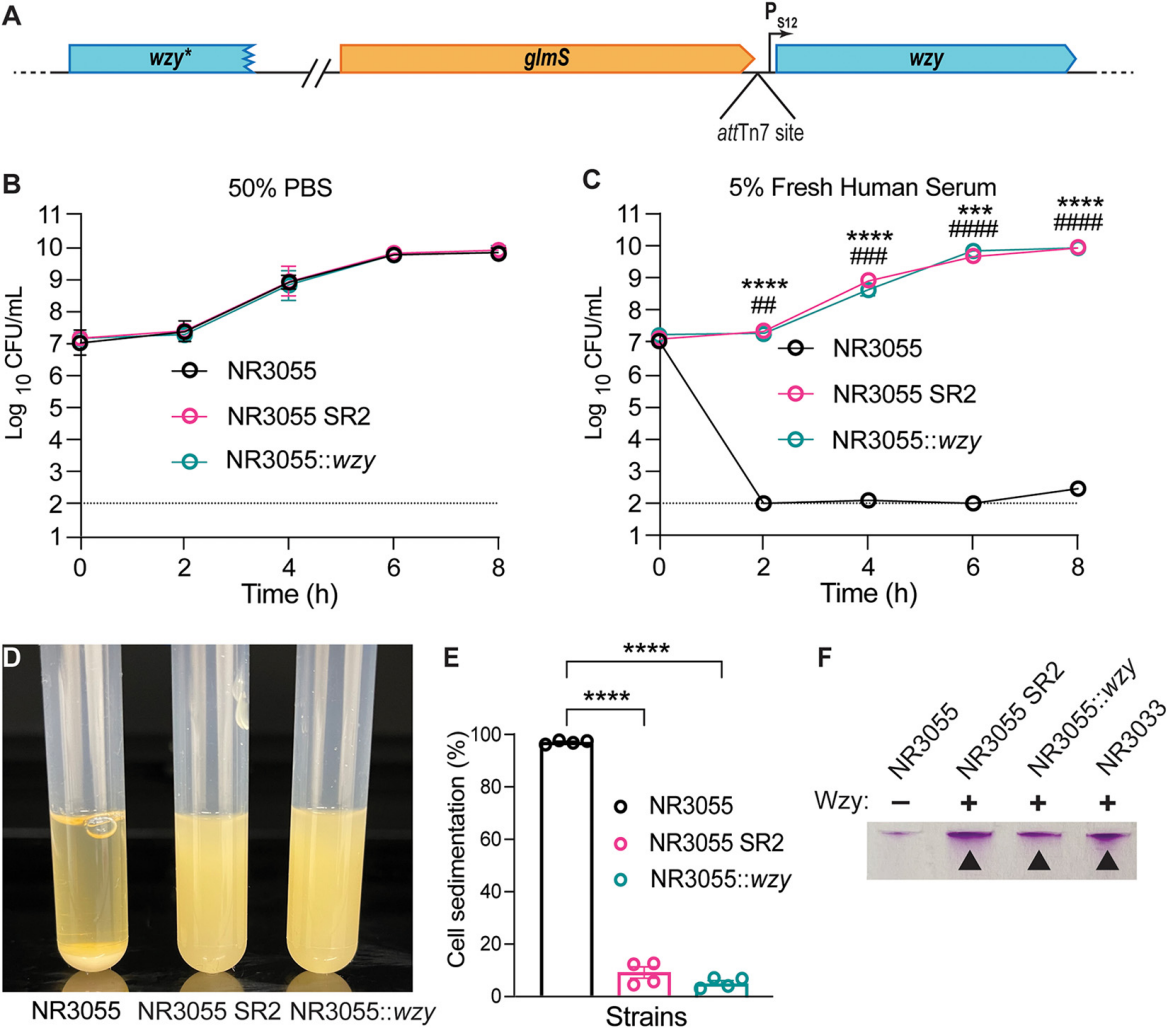
Expression of full-length *wzy* confers serum resistance to NR3055. (A) Schematic detailing the genetic complementation of *wzy* in NR3055. Wild-type *wzy* was expressed from the constitutive P_S12_ promoter at the neutral *att*Tn7 site on the NR3055 chromosome, downstream of *glmS*. The natural, truncated *wzy* gene on the NR3055 chromosome is labeled as wzy*. (B and C) NR3055, NR3055 SR2, and NR3055*::wzy* were cultured in either 50% TSB + 50% PBS (B) or 50% TSB + 45% PBS + 5% fresh pooled human serum (C). The cultures were diluted in PBS and plated on TSA for CFU every 2 h for 8 h. The limit of detection for this assay is 100 CFU/mL (dotted lines). Each strain is represented by three biological replicates. The error bars indicate the SEM. Statistical significance was determined via a two-way ANOVA with Dunnett’s multiple-comparison test. Asterisks represent NR3055 compared to NR3055 SR2, and pound signs represent NR3055 compared to NR3055::*wzy* (****/####, *P ≤ *0.0001; ***/###, *P ≤ *0.001; **/##, *P ≤ *0.01). (D and E) NR3055 cells, but not NR3055 SR2 or NR3055::*wzy* cells, sediment in liquid culture. Sedimentation is demonstrated visually (D) and quantitatively (E). The percent cell sedimentation was calculated using the inverse ratio of the OD_600_ of the mixed culture to the OD_600_ of the same culture after a 4 h period of stagnant incubation. Strains are represented by four biological replicates. The error bars indicate the SEM. Statistical significance was determined via a one-way ANOVA with Dunnett’s multiple-comparison test (****, *P ≤ *0.0001). (F) The extracellular polysaccharide content from NR3055, NR3055 SR2, NR3055::*wzy*, and NR3033 was separated using Tris-acetate protein gels and polysaccharides stained with a Pierce Glycoprotein Staining Kit. NR3033 is a ST78, *cps* type NL68 *E. hormaechei* strain representing “wild-type” CPS production for ST78. Arrows demark the pink high molecular weight bands that are representative of CPS. A representative gel is shown. The extracellular polysaccharide content of the three other identified serum sensitive strains can be seen in Fig. S3.

To investigate whether the premature stop codon in *wzy* is the reason for the serum-sensitive phenotype of NR3055, we complemented NR3055 with wild-type (WT) *wzy*, resulting in strain NR3055 *att*Tn7::P_S12_-*wzy* (referred to as NR3055::*wzy*) (Fig. 6A). Whereas NR3055, NR3055 SR2, and NR3055::*wzy* grew equally well in TSB with 50% PBS (Fig. 6B), the addition of 5% fresh human serum only affected the growth of parental NR3055, killing this strain by 5 orders of magnitude within the first 2 h of culture. Conversely, NR3055 SR2 and NR3055::*wzy* displayed normal, uninhibited growth in 5% fresh human serum (Fig. 6C).

10.1128/mbio.02590-22.3 FIG S3 Extrapolysaccharide content from serum sensitive strains and their comparators. The extracellular polysaccharide content from NR3055, NR3055 SR2, NR3055::*wzy*, NR3033, NR3082, NR2980, KP1593, and KP0589 was separated using Tris-acetate protein gels and polysaccharides stained with a Pierce Glycoprotein Staining Kit. NR3033 is an ST78, CPS type NL68 *E. hormaechei* strain representing “wild-type” CPS production for ST78. NR3082, KP1593, and KP0589 are known serum sensitive strains and show no CPS production. NR2980 is a closely related ST171 isolate to NR3082 and serves as its comparator. No comparators for KP1593 or KP0589 are available in our isolate collection. Arrows demark the pink high molecular weight bands that are representative of CPS. A representative gel is shown. Download FIG S3, PDF file, 0.9 MB. Copyright © 2023 St. John et al. 2023 St. John et al. https://creativecommons.org/licenses/by/4.0/ This content is distributed under the terms of the Creative Commons Attribution 4.0 International license.

When cultured in broth, NR3055 cells from stationary-phase cultures sedimented to the bottom of the culture tube when left stagnant, whereas NR3055 SR2 and NR3055::*wzy* remained in suspension (Fig. 6D). The average percent cell sedimentation values of NR3055, NR3055 SR2, and NR3055::*wzy* were 97%, 8.6%, and 5%, respectively (Fig. 6E), as measured by the optical density. These data supported our hypothesis that the premature stop codon in the *wzy* gene of NR3055 abrogated CPS production, as cell surface-associated polysaccharides (e.g., lipopolysaccharide and CPS) are known to affect cellular buoyancy (32, 33).

To directly evaluate CPS levels, extracellular polysaccharide was extracted from NR3055, NR3055 SR2, and NR3055::*wzy*. The samples were separated on a gel, and the polysaccharides were stained. These data extended the TEM observations (Fig. 4A and B) and showed that NR3055 SR2 and NR3055::*wzy* indeed have more polysaccharides/CPS on their surfaces, compared to NR3055 (Fig. 6F).

### NR3055 SR2 and NR3055::*wzy* are virulent in a murine peritonitis model.

**FIG 7:**
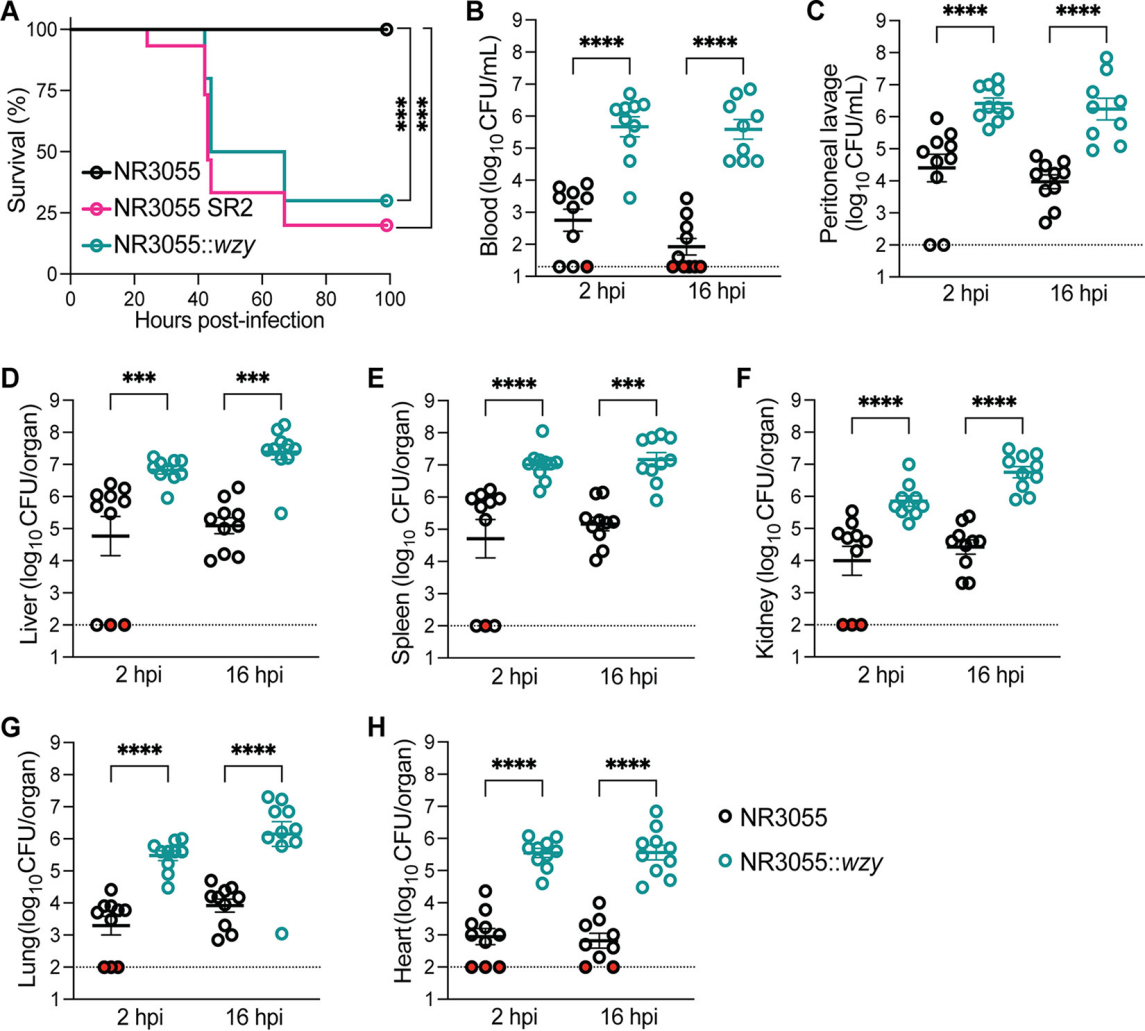
The *wzy*-dependent virulence of NR3055 in a murine peritonitis model. (A) Mice were infected intraperitoneally (i.p.) with 10^8^ CFU of NR3055, NR3055 SR2, or NR3055::*wzy* and monitored for survival. The morbidity of the animals was monitored for 4 days. Statistical analysis was performed via a log-rank (Mantel-Cox) test that was corrected for multiple comparisons. The data are from at least two independent experiments with a total of 10 mice per group (***, *P* < 0.001). (B–H) Mice were infected i.p. with 10^8^ CFU of NR3055 or NR3055::*wzy*, and at 2 and 16 h postinfection (hpi), blood was collected via cardiac puncture (B). Peritoneal lavages were also collected (C), and the indicated organs were harvested (D–H). The organs were homogenized, and the blood, peritoneal lavage fluid, and organ homogenates were serially diluted in PBS and plated for CFU. The limit of detection for blood bacterial burdens is 20 CFU/mL, and that of all other organs and peritoneal lavage fluid is 100 CFU/mL (dotted lines). Red-filled circles indicate no recovered CFU. The error bars indicate the SEM. Each circle represents an individual mouse. The data are from two independent experiments, with a total of 10 mice per group. Statistical significance was determined via a one-way ANOVA with Šidák’s multiple-comparison test (****, *P ≤ *0.0001; ***, *P ≤ *0.001).

Next, we investigated the contribution of CPS to *E. hormaechei* pathogenesis by using a murine model of peritonitis (34). C57BL/6J mice were infected via an intraperitoneal (i.p.) injection containing a lethal dose of approximately 10^8^ CFU of NR3055, NR3055 SR2, or NR3055::*wzy*, and the animals were monitored for morbidity and survival. While we observed that NR3055 was avirulent in this model, NR3055 SR2 was highly virulent, a phenotype that was also observed with NR3055::*wzy* (Fig. 7A).

We also measured the bacterial burdens in the blood, peritoneal lavage fluid, spleen, kidneys, liver, lungs, and heart at 2 h and 16 h postinfection (hpi). At both time points, the mice infected with NR3055::*wzy* had significantly higher bacterial burdens than did the mice infected with NR3055 across all tissues (Fig. 7B–H). Collectively, these data indicate that functional Wzy is required for the virulence and dissemination of *E. hormaechei*.

### The *E. hormaechei* CPS acts as a protective barrier against the innate immune response.

**FIG 8:**
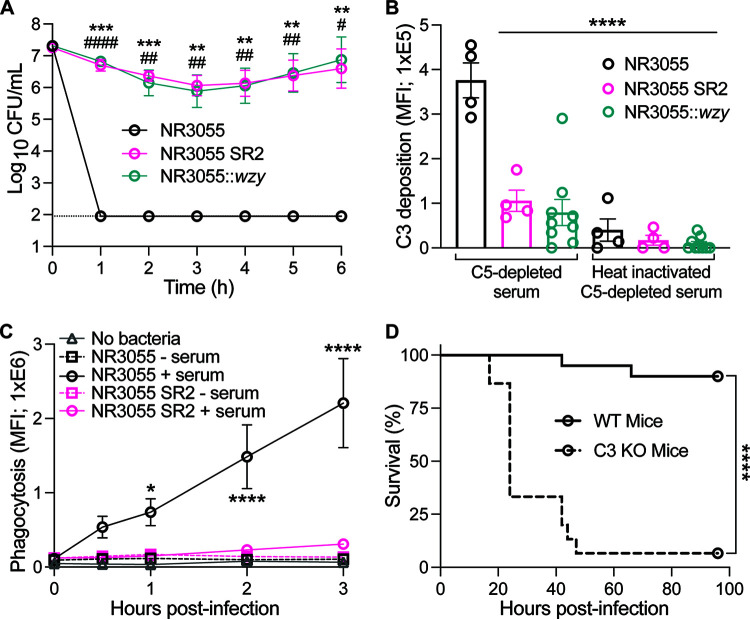
The *wzy*-dependent evasion of innate immune defenses. (A) NR3055, NR3055 SR2, and NR3055::*wzy* (2 × 10^7^ CFU/mL) were inoculated in whole human blood, and cultures were serially diluted in PBS and plated for CFU every hour for 6 h. Each strain is represented by two biological replicates, with two separate human blood donors per replicate. The limit of detection for this assay is 90 CFU/mL (dotted line). The error bars indicate the SEM. Statistical significance was determined via a two-way ANOVA with Dunnett’s multiple-comparison test. Asterisks represent NR3055 compared to NR3055 SR2, and pound signs represent NR3055 compared to NR3055::*wzy* (****/####, *P ≤ *0.0001; ***/###, *P ≤ *0.001; **/##, *P ≤ *0.01; */#, *P ≤ *0.1). (B) C3b deposition on the surface of NR3055, NR3055 SR2, and NR3055::*wzy* was analyzed by measuring the mean fluorescence intensity (MFI) via flow cytometry, using an anti-complement C3b/iC3b antibody. The data are from three experimental days of either one, three, or five biological replicates. The error bars indicate the SEM. Statistical significance was determined via a one-way ANOVA with Dunnett’s multiple-comparison test. All of the experimental groups are compared to NR3055 (****, *P ≤ *0.0001). (C) Primary human neutrophils were infected at a multiplicity of infection (MOI) of 10 with either opsonized or nonopsonized GFP-producing NR3055 or NR3055 SR2, or buffer alone as a control. The bacterial uptake over time is represented by the MFI and was determined via flow cytometry. The data are representative of two biological replicates with two human blood donors per replicate. The error bars indicate the SEM. Statistical significance was determined via a two-way ANOVA with Dunnett’s multiple-comparison test (****, *P ≤ *0.0001; *, *P ≤ *0.1). All of the experimental groups are compared to NR3055 opsonized with 2% serum. Asterisks represent all comparisons at each time point. (D) 20 wild-type (5 male and 15 female) and 15 C3-deficient (6 male and 9 female) 8-week-old C57BL/6J mice were infected with NR3055. All male mice were infected with 2.5 × 10^8^ CFU, and all female mice were infected with 10^8^ CFU. All mice were monitored for morbidity and survival for 4 days. The statistical analysis was performed via a log-rank (Mantel-Cox) test that was corrected for multiple comparisons. The data are from at least two independent experiments with a total of *n* = 15 to 20 mice per group (****, *P* ≤ 0.0001).

To further explore the impact of Wzy and CPS production on the pathogenesis of *E. hormaechei*, we employed a human whole blood model. When grown in whole-human-blood, NR3055::*wzy* phenocopied NR3055 SR2, whereas the viability of NR3055 decreased to the limit of detection within 1 h of culture (Fig. 8A).

A critical early step in complement-mediated killing is the deposition of C3b on the bacterial cell surface. To test whether NR3055::*wzy* would prevent C3b deposition on its surface, we measured C3b binding to cells as described previously (19). We observed that NR3055 had significantly increased C3b deposition on the cell surface, compared to NR3055 SR2 and NR3055::*wzy* (Fig. 8B).

The deposition of C3b is also critical for the opsonization of bacterial cells. Thus, we hypothesized that NR3055 SR2 could also evade phagocytosis by neutrophils via the Wzy-dependent inhibition of C3b binding. To test this, green fluorescent protein (GFP)-producing NR3055 and NR3055 SR2 strains were used to infect primary human neutrophils (PMNs), and the uptake by PMNs (including PMN-associated and -phagocytosed bacteria) was measured. These data demonstrate that NR3055 SR2 evaded uptake by PMNs, whereas NR3055 exhibited an opsonization-dependent uptake by PMNs that steadily increased over time (Fig. 8C).

The data above suggest that complement is a key antimicrobial defense against *E. hormaechei*. We reasoned that a host that is defective in complement is likely susceptible to unencapsulated isolates. To directly test this, we compared the susceptibility of WT and C3-deficient mice to NR3055. Of the NR3055-infected C3^−/−^ mice, 93% succumbed to infection within 50 hpi, compared to only 10% of the WT mice (Fig. 8D). Together, these data demonstrate that CPS is an important *E. hormaechei* virulence factor that is responsible for the evasion of complement during infection.

### Recurrent infection with CPS-producing *E. hormaechei* provides protection against future lethal infections.

**FIG 9:**
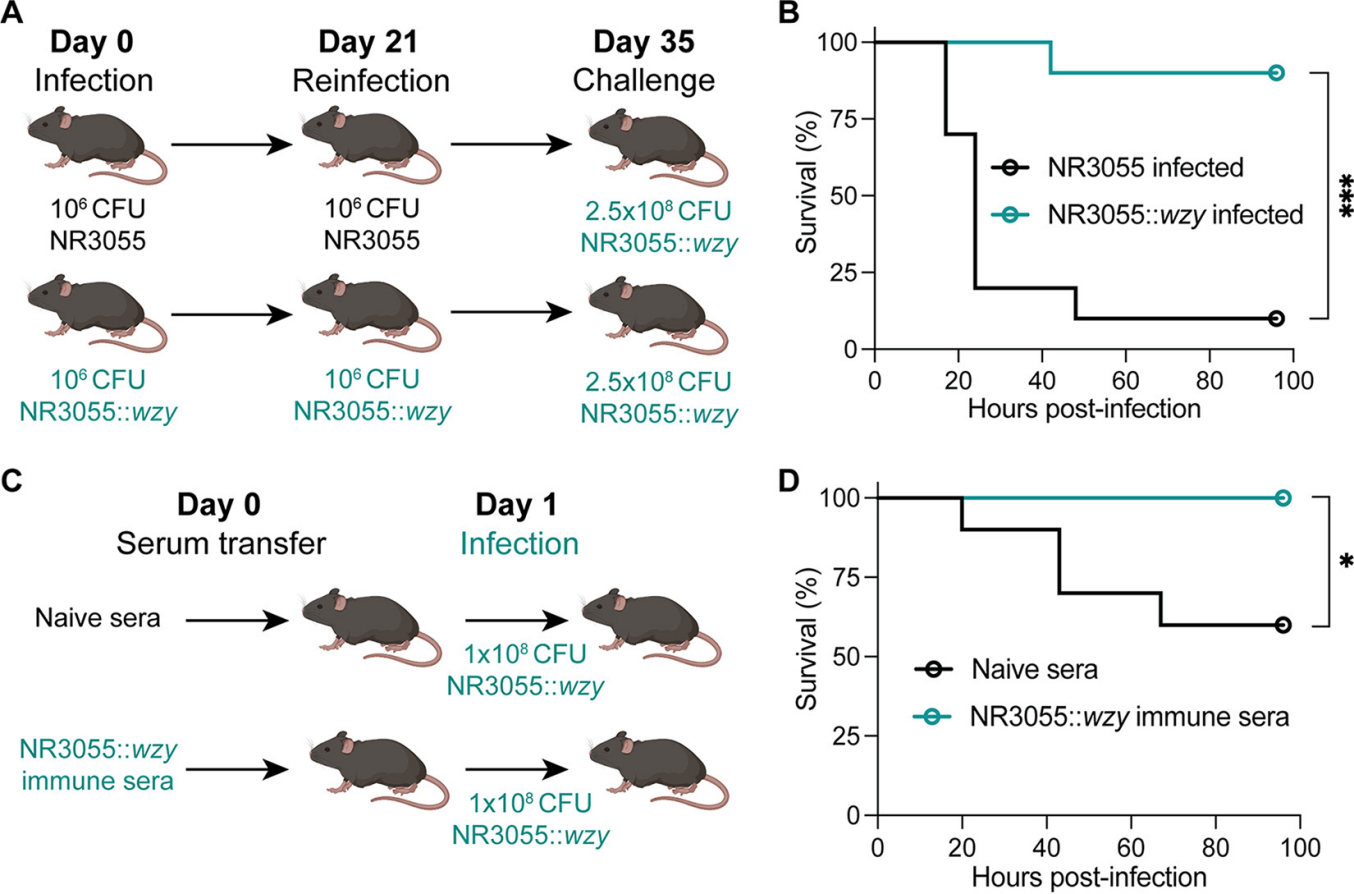
CPS-producing NR3055::*wzy* provides immunity against future infections. (A) Diagram of the experimental design. Mice were infected intraperitoneally (i.p.) with 10^6^ CFU of NR3055 or NR3055::*wzy*. Three weeks postinfection, the mice were reinfected i.p. with 10^6^ CFU of NR3055 or NR3055::*wzy.* Two weeks post the second exposure, all mice were challenged with 2.5 × 10^8^ CFU of NR3055::*wzy* and monitored for morbidity and survival for four days. (B) The mice were infected and monitored as detailed in panel A. The statistical analysis was performed via a log-rank (Mantel-Cox) test. The data are from two independent experiments, with a total of 10 mice per group (***, *P* < 0.001). (C) Diagram of the experimental design. The mice that survived the lethal challenge with NR3055::*wzy* (B) were terminally bled, and their sera were collected and pooled. Naive mice were then passively immunized with either serum from the NR3055::*wzy*-infected mice or with serum from uninfected naive control mice. The mice were then infected 24 h post passive transfer with 10^8^ CFU of NR3055::*wzy* and monitored for morbidity and survival for four days. (D) The mice were infected and monitored as detailed in panel C. The statistical analysis was performed via a log-rank (Mantel-Cox) test. The data are from two independent experiments. with a total of 10 mice per group (*, *P ≤ *0.1). Panels A and C were created using BioRender.com.

Due to the critical role of CPS for *E. hormaechei* pathogenesis (Fig. 7 and 8), we next investigated whether the host could mount an anti-CPS response to protect itself from infection. To this end, we employed a recurrent infection model in which mice were infected intraperitoneally with a nonlethal dose (10^6^ CFU) of NR3055 or NR3055::*wzy* twice, with a three week recovery period between the infections (Fig. 9A). The mice were then challenged with a lethal dose (2.5 × 10^8^ CFU) of NR3055::*wzy* two weeks after the second exposure, and they were monitored for morbidity and survival. Of the mice that were preinfected with the unencapsulated NR3055 strain, 90% succumbed to infection with the encapsulated NR3055::*wzy* strain, whereas almost all of the mice that were preinfected with the encapsulated NR3055::*wzy* strain were protected from the lethal challenge (Fig. 9B).

To test whether the observed protection was transferable by serum, we isolated serum from the mice that survived the NR3055::*wzy* recurrent infections and performed passive immunization studies into naive mice (Fig. 9C). Compared to mice that received serum that was isolated from unexposed mice, passive immunization with serum from mice that were previously exposed to NR3055::*wzy* conferred protection from lethal NR3055::*wzy* infection (Fig. 9D). These findings suggest that the host can mount an immune response against the *E. hormaechei* CPS that is protective against lethal infections.

## DISCUSSION

*E. hormaechei* is a prominent ECC species of clinical concern due to its increasing antimicrobial-resistance and high mortality rates in vulnerable patient populations (9, 35). The molecular mechanisms promoting *E. hormaechei* pathogenesis and its evasion of the host immune response are poorly understood. By employing a population-based screen of Enterobacter spp. clinical isolates to assess their susceptibility to the bacteriostatic and bactericidal activities of human serum, we identified isolates that were severely serum sensitive. It is not surprising that the vast majority of the isolates in our collection exhibit some degree of resistance to human serum, given that *E. hormaechei* and other ECC pathogens notoriously cause infections in multiple tissues (1) and must withstand the antibacterial activity of serum to survive and disseminate to these body sites. Our screen identified four ECC isolates that exhibited severe serum sensitivity: NR3082, KP1593, KP0589, and NR3055. To dissect the mechanism(s) of serum resistance, we focused on one isolate, NR3055. Our genomic analysis of KP0589 identified an integrative plasmid in the center of its *cps* locus, which likely results in decreased CPS biosynthesis and serum sensitivity. We have not uncovered the genetic bases for the serum sensitive phenotypes of NR3082 or KP1593; however, there is little to no extracellular polysaccharide content associated with these isolates when visualized via a glycoprotein stain (Fig. S3). Future experiments will investigate the genetic determinants for the serum susceptibilities of NR3082, KP1593, and KP0589. We combined *in vitro*-based selection (Fig. 3), comparative genomics (Fig. 5), and complementation studies (Fig. 6) with human whole blood (Fig. 8A) and preclinical *in vivo* murine models (Fig. 7 and 9) to establish the role of Wzy-dependent CPS biosynthesis in *E. hormaechei* pathogenesis. Collectively, our data support a model in which *E. hormaechei* uses CPS to subvert complement-mediated immunity, promoting infection and bacterial dissemination.

Regarding how serum-sensitive ECC isolates could survive *in vivo* during infections of humans, ECC pathogens commonly cause infections in immunocompromised individuals (1), which could enable an otherwise attenuated pathogen to thrive *in vivo*. Consistent with this notion, the studies using C3-deficient mice (Fig. 8D) demonstrate that NR3055, a CPS-deficient strain, is virulent when infecting a host that lacks the complement cascade (Fig. 8D). Additionally, isolates from hospital-associated infections are well-known to exhibit low virulence and genome degradation (36).

Given that ECC infections are becoming increasingly recalcitrant to standard antibiotic therapy, elucidating the mechanisms that are used by these pathogens to cause infection is paramount for the development of new, effective therapeutic and prophylactic interventions. CPS functions as a virulence factor in both Gram-negative and Gram-positive pathogens (37–43). In fact, vaccines targeting CPS have resulted in extremely effective strategies against clinically relevant pathogens, such as Streptococcus pneumoniae, Neisseria meningitidis, and Haemophilus influenzae serogroup B (44–47). Our recurrent infection and passive immunization data (Fig. 9) demonstrate that the *E. hormaechei* CPS is also targetable and that the host mounts a protective humoral immune response that could possibly be exploited for the development of future vaccines and/or biologics. We postulate that host-derived, anti-CPS antibodies overcome *E. hormaechei* immune evasion by enabling complement deposition, which in turn promotes neutrophil-mediated opsonophagocytic killing and pathogen clearance. Future studies are needed to test the immunogenicity of purified *E. hormaechei* CPS and to establish the molecular mode of protection.

In conclusion, we describe here an in-depth, mechanistic study of Enterobacter pathogenesis and establish CPS as a potential target to combat these important pathogens. Future studies that continue to bridge the gap in our knowledge of the pathogenesis and the host immune response to Enterobacter pathogens are needed to provide crucial information that can be leveraged for the design of much needed therapeutics for the prevention of or intervention against these high-priority ESKAPE pathogens.

## MATERIALS AND METHODS

### Ethics statement.

Human blood samples were obtained as buffy coats from healthy, anonymous, consenting adult donors (New York Blood Center). Freshly isolated human whole blood was collected in accordance with a protocol approved by the NYU Grossman School of Medicine Institutional Review Board for Human Subjects (Torres Lab IRB number number i14-02129_CR6). All donors provided written consent to participate in the study.

All of the experiments involving animals were reviewed and approved by the Institutional Animal Care and Use Committee of NYU Langone Health and were performed according to guidelines from the National Institutes of Health (NIH), the Animal Welfare Act, and US Federal Law.

### Bacterial strains, plasmids, and growth conditions.

All of the bacterial strains and plasmids used in this study are listed in Tables S1 and S3. Enterobacter isolates were routinely cultured in tryptic soy broth (TSB) with aeration at 37°C or on tryptic soy agar (TSA) at 30°C, with the media being supplemented with 50 μg/mL chloramphenicol when appropriate (when culturing on TSA with chloramphenicol, plates were incubated at 37°C). Escherichia coli strains were routinely cultured in lysogeny broth (LB) with aeration at 37°C or LB agar at 37°C, with the media being supplemented with 34 μg/mL chloramphenicol or 100 μg/mL ampicillin when appropriate.

10.1128/mbio.02590-22.6 TABLE S3 Strains and plasmids used in this study. Download Table S3, DOCX file, 0.01 MB. Copyright © 2023 St. John et al. 2023 St. John et al. https://creativecommons.org/licenses/by/4.0/ This content is distributed under the terms of the Creative Commons Attribution 4.0 International license.

### Serum susceptibility screen of Enterobacter clinical isolates.

Residual clinical isolates of Enterobacter spp. were collected in New York City, after the completion of a diagnostic process (from routine AMR surveillance by the Mount Sinai Health System Pathogen Surveillance Program [*n *= 4] [IRB approved, HSnumber 13-00981]), as a part of the NYU Langone Health COVID-19 superinfection biorepository (*n *= 23), and isolates from the Columbia University Medical Center (*n *= 69), which were previously reported (17), were examined for their abilities to grow in human serum. 96 isolates were grown overnight in a 96-well plate from a single colony. The next day, the overnight cultures were diluted 1:100 (990 μL TSB, 10 μL overnight) in a deep-well plate. In a 96-well plate, 6 μL of the diluted overnight culture was added to 181.5 μL TSB. In a honeycomb plate, 62.5 μL of culture was added to either 62.5 μL PBS or 62.5 μL pooled human serum (SeraCare Life Sciences, Inc., Milford, MA). The control wells included 50% TSB + 50% PBS alone and 50% TSB + 50% pooled human serum. Using a BioScreen C Automated Microbiology Reader, the Enterobacter spp. isolates were grown at 37°C, and the OD_600_ of each well was taken every 30 min for 24 h.

### Preparation of fresh human serum.

Blood was drawn from 11 consenting, healthy individuals into BD Vacutainer Serum Tubes (Becton, Dickinson and Company, Franklin Lakes, NJ). The blood was allowed to clot at room temperature for approximately 20 min. After clotting, the tubes were kept on ice to preserve complement activity. The tubes were centrifuged at 3,000 rpm for 10 min at 4°C. Keeping the tubes on ice, the serum was collected in 50 mL conical tubes. The tubes were centrifuged again at 3,000 rpm for 10 min at 4°C so that the remaining erythrocytes would pellet. The serum from all donors was pooled and aliquoted in microcentrifuge tubes. The serum was stored at –80°C.

### Human serum killing screen of Enterobacter clinical isolates.

Of the 96 Enterobacter spp. clinical isolates, 62 were tested in a serum bactericidal activity assay. Overnight cultures were diluted in TSB to a cell concentration of approximately 2 × 10^7^ CFU/mL. The diluted overnight cultures were then further diluted 1:1 with either PBS or pooled human serum (SeraCare Life Sciences, Inc.), resulting in starting cell concentrations of approximately 10^7^ CFU/mL. For the experiments using 5% fresh human serum, the diluted overnight cultures were then further diluted either 1:1 with PBS or 1:1 with a 10% fresh human serum/90% PBS mixture. The cultures were incubated at 37°C with shaking at 180 rpm. Every 2 h, over an 8 h period of time, aliquots from the cultures were serially diluted in PBS, and plated on TSA for CFU. A killing curve was generated by plotting the CFU/mL values over time.

### NR3055 serum-resistant clone selection.

15 cultures of NR3055 were set up from single colonies in 2 mL TSB and grown overnight at 37°C and 180 rpm. The next day, a sterile cotton swab was used to liberally spread the overnight cultures on separate TSA plates so that a lawn of bacteria would grow. Next, 300 μL of pooled human serum (SeraCare Life Sciences, Inc.) was spotted in the center of the plate on which the bacteria had been swabbed. The plates were incubated overnight at 30°C. The following day, the serum had formed a zone of killing, although there were several colonies that grew within that zone. These colonies were potentially serum-resistant clones of NR3055. These colonies were picked and inoculated into individual single wells of a 96-well plate that contained 200 μL TSB. The 96-well plate was grown overnight at 37°C and 180 rpm. The next day, each well was swabbed onto a TSA plate, and the bacteria were spotted with 50 μL SeraCare pooled human serum. The plates were incubated overnight at 30°C. For clones that did not have a zone of killing, cells were collected from the plate and frozen in LB + 20% glycerol to later be confirmed to be serum-resistant through a serum killing assay and be tested in a whole blood killing assay.

### Whole human blood killing.

Whole human blood was collected from consenting volunteers into BAPA (benzylsulfonyl-d-Arg-Pro4-amidinobenzylamide) anticoagulant blood collection tubes (cat. number 18004, Diapharma Group, Inc., West Chester, OH). Bacteria were inoculated into the whole human blood at a starting concentration of approximately 2 × 10^7^ CFU/mL. Cultures were incubated at 37°C in a roller drum incubator. Every hour, for 6 h, aliquots were taken and incubated with 0.1% saponin on ice for 20 min. The samples were serially diluted in PBS and plated for CFU on TSA. A killing curve was generated by plotting the CFU/mL values versus time.

### Transmission electron microscopy.

NR3055 and NR3055 SR2 were visualized by the NYU Langone Health Microscopy Laboratory via high pressure freezing and freeze-substitution transmission electron microscopy (TEM), following a previously established protocol (48). Briefly, cultures were grown overnight in 2 mL TSB at 37°C and 180 rpm. Following growth, 1 mL samples of stationary-phase cells were placed on ice and centrifuged at a low speed (3,000 rpm) for 10 min, and the pellets were gently resuspended in 20% bovine serum albumin (BSA). Planchette hats with 100 μm deep wells were lightly coated with hexadecene before being filled with approximately 1.2 μL of bacterial sample. Flat sided hats were also lightly coated with hexadecene and placed on top of the welled hats. The hats were sealed in the planchette holder, and high pressure freezing commenced, using a Wohlwend Compact HPF-01 High Pressure Freezer at the New York Structure Biology Center. The frozen hats were immediately transferred from the planchette holder into liquid nitrogen. The samples were transferred into cryovials containing a mixture of 2% osmium tetroxide and 2% (wt/vol) uranyl acetate in anhydrous acetone with 0.075% (wt/vol) ruthenium red in acetone at liquid nitrogen temperature (freeze substitution solution) and then brought into a Leica freeze substitution unit. Since the acetone:osmium mixture liquifies at –90°C, the hats were slowly submerged into the freeze substitution media. The samples were kept at –90°C for 20 h. Freeze substitution continued by increasing the temperature for 3 h to –60°C. The temperature was held at –60°C for 10 h, and this was followed by another 3 h increase to −30°C, holding at −30°C for 20 h, and a final increase for 5 h to room temperature, which was held for 11 h. Three 1 h exchanges of pure acetone were used to rinse out the osmium. Infiltration at room temperature began with a 1:1 mixture of acetone:epon for >1 h, and it continued overnight with a 1:2 acetone:epon mixture. The samples were allowed to sit in pure Embed 812 (Electron Microscopy Sciences, Harfield, PA) for >4 h before embedment in embedding blocks. The samples were cured at 60°C for 48 h. 70 nm thin sections of the cells were mounted on 200 mesh EM grids and stained with uranyl acetate and lead citrate, using standard methods. The stained grids were imaged on a Talos L120C transmission electron microscope (Thermo Fisher Scientific, Hillsboro, OR) with a Gatan 4k × 4k OneView camera (Gatan Inc. Pleasanton, CA).

To quantify the width of CPS that was produced by NR3055 and NR3055 SR2, the TEM images were edited in Fiji (49). Cell diameters were measured in pixels and are indicated with cyan lines, whereas capsule widths were measured in pixels and are indicated with magenta lines (Fig. S2). Four capsule-width measurements were taken per cell image. The capsule widths were averaged per image and normalized to the cell diameter.

### Sequencing, assembly, and annotation of bacterial genomes.

Newly collected isolates underwent Illumina sequencing, and we resequenced the isolates from Columbia University Medical Center (17) to ensure equal coverage. Genomic DNA was isolated from 96 Enterobacter clinical isolates and 8 NR3055 SR clones using a Wizard Genomic DNA Purification Kit (Promega Corporation, Madison, WI), following the manufacturer’s protocol for isolating genomic DNA from Gram-negative bacteria. Bacterial whole-genome library preparation was performed using a Nextera DNA Flex Library Prep Kit (96rxn kit, cat. number 20025520, Illumina, San Diego, CA). DNA concentrations were verified using a Quant-iT High Sensitivity dsDNA Assay Kit (cat. number Q33120, ThermoFisher, Waltham, MA) and a microplate reader. The library preparation is a bead-based tagmentation reaction that cuts the dsDNA (100 ng input, each normalized to 30 μL volume) to roughly 350 bp in size. All subsequent library preparation steps are done on the beads. The protocol was modified to include the use of one-quarter scale reaction mixes for all reactions throughout the library preparation procedure. Following PCR amplification (five cycles in total), according to the protocol, water was added (38 μL) to the amplified material (12.5 μL) to raise the volume up to 50 μL for the final 1× Ampure XP bead cleanup (cat. number A63882, Beckman Coulter Life Sciences, Indianapolis, IN). Library DNA was run on a TapeStation 2200 with high sensitivity DNA screentape (Agilent Technologies, Santa Clara, CA) to verify a library size of approximately 500 bp to 650 bp, and each individual library was run on qPCR for library quantitation using a Kapa-Roche Library Quant Kit (cat. number KK4824, Roche) on a CFX384 Touch Real-Time PCR Detection System (Bio-Rad Laboratories, Hercules, CA). Sequencing was run on a NovaSeq 6000 System (Illumina, San Diego, CA), using a full SP300 cartridge and flow cell (paired end 150 dual indexing run), which resulted in approximately 250× coverage. Illumina sequencing runs were preprocessed with fastp v. 0.20.1 (50), using the default parameters. Preprocessed sequencing runs were controlled for interspecies and intraspecies contamination using confindr v. 0.7.4 (51) and metaPhlAn v. 3.0.13. (52). The genomes of strains for which solely Illumina sequencing was available were assembled using Unicycler v. 0.4.8 (53) with the default parameters after subsampling the read sets to 120× coverage using bbnorm v. 38.92 with the *tossbadreads* flag (https://sourceforge.net/projects/bbmap/).

The genomes of NR3055 and NR3055 SR2 were also long-read sequenced using Oxford Nanopore Technologies (ONT). High molecular weight genomic DNA was purified from strains using a MasterPure Complete DNA & RNA Purification Kit (Lucigen Corporation, Middleton, WI), following the manufacturer’s protocol. Library preparation was performed following the ONT Native Barcoding Genomic DNA protocol, using the Native Barcoding Expansion 1 to 12 (EXP-NBD104) and Ligation Sequencing Kit (SQK-LSK109). The sequencing library was run on the ONT MinION platform, using the flow cell R9.4.1. Quality filtering was carried out using Filtlong v0.2.0 (https://github.com/rrwick/Filtlong). The genomes of strains for which ONT sequencing was available were assembled, long reads first, using Trycycler v. 0.4.1 (54). The ONT reads were randomly subsampled into 12 read sets, of which one-third were assembled with flye v. 2.8.3-b1695, one-third with raven v. 1.5.0, and one third with the miniasm_and_minipolish.sh script that was provided with minipolish v. 0.1.2 (55), using miniasm v. 0.3-r179 (56). After running the “cluster”, “reconcile”, “msa”, “partition”, and “consensus” commands of trycycler, using the default parameters, the assemblies were polished, using the long reads with medaka v.1.4.3 (https://github.com/nanoporetech/medaka) and specifying the r941_min_hac_g507 error correction model. Finally, the assemblies were polished by aligning the Illumina short reads to them by using bwa mem 0.7.17 (57) and by processing the alignments with pilon v. 1.24, using the default parameters and the –frags parameter for alignment input, iterating alignment and pilon polishing until pilon did not make further improvements to the assembly, for a maximum of 10 iterations. To ensure that the ONT coverage was sufficient to capture all replicons, we additionally hybrid-assembled with both Illumina and ONT read sets, using Unicycler v. 0.4.8 (53) with the default parameters. Contigs from the Unicycler and Trycycler assemblies were compared using dnadiff v. 1.3 (58) and mummerplot 3.5. The best set of contigs for each isolate was manually selected. Specifically, circularized contigs from the long-read assemblies were retained, as these did not feature the large collapsed regions that we had identified in the hybrid assemblies, but contigs that were missing from the long-read assemblies due to low coverage were obtained from short-reads-first hybrid assemblies.

The assemblies were annotated with the species, MLST, capsule type, and genes, as follows. The taxonomical lineage was identified using GTDB-Tk v1.5.0 (59) with reference data version r202. Using mlst v. 2.19.0 (https://github.com/tseemann/mlst) and the PubMLST (60) scheme for Enterobacter cloacae, STs were assigned to assemblies. Capsule typing was carried out with Kaptive v0.7.3 (61), using an Enterobacteriaceae *cps* database (29). Within this database, a gene call for *ugd* within the Enterobacter-NL68 locus was found to be missing and was transferred from the RefSeq (62) assembly NZ_CP034754.1. Capsule typing results with a match confidence of “None” or “Low” were disregarded, whereas those with a match confidence of “Perfect”, “Very high”, “High” or “Good” were included. The genes were annotated using PGAP v. 2021-11-29.build5742 (63).

### Comparative genomics.

All 3,014 of the available Enterobacter assemblies were downloaded from RefSeq (62) on October 26, 2021. Of those, 1,886 assemblies were identified to be Enterobacter hormaechei *A* by GTDB-Tk (see “Sequencing, assembly, and annotation of bacterial genomes”) (Table S2). The remaining 1,128 assemblies were disregarded. MLST and capsule types were assigned as described above, but only 979 assemblies could be assigned both a sequence and a capsule type.

Genome-wide single nucleotide variants (SNVs) and indels were identified using Illumina sequencing data and the snippy v. 4.6.0 pipeline (https://github.com/tseemann/snippy), using our NR3055 assembly as a reference. This pipeline was also used to compute two core-genome alignments for phylogenetic tree construction. One core-genome alignment was constructed with the nucleotides of all 104 of the isolates that were sequenced in this study (1,736,091 alignment positions, of which 301,895 were variable). The other core-genome alignment was comprised of nucleotides from all of the ST78 strains from this study as well as those downloaded from RefSeq, but these only included those with a sufficiently confident *cps* locus type (see “Sequencing, assembly, and annotation of bacterial genomes”) (Tables S1 and S2) (4,087,917 alignment positions, of which 4,167 were variable). The phylogenies were computed with RAxML v. 8.2.12 (64), using the GTRGAMMA model, 100 rapid bootstrap replicates, and a search for best-scoring maximum likelihood tree.

To obtain variants within the *cps* loci of the ST78 *E. hormaechei* assemblies, we first extracted the *cps* loci from the assemblies by using the reference Enterobacter-NL68 *cps* locus (see “Sequencing, assembly, and annotation of bacterial genomes”) as a query and blastn v. 2.11.0 (65) with the default parameters. We then performed a nucmer (58) alignment of the extracted *cps* loci to the entire *cps* locus reference as well as to each individual gene within the locus reference. We ran programs from the mummer (58) suite via the pymummer wrapper (https://github.com/sanger-pathogens/pymummer), specifying a minimum identity of 90%, a minimum length of 20, a break length of 200, and the *maxmatch* and *show_snps* flags. We extracted variants within genes by using the individual gene alignments and intergenic variants within the *cps* locus, using the alignment to the entire locus reference.

### Complementation of *wzy* in NR3055.

The WT *wzy* gene was PCR amplified from the serum-resistant clone NR3055 SR2 and cloned into the pUC18R6KS12Cm vector. We generated pUC18R6KS12Cm by using the pUC18R6K-mini-Tn7T-Gm backbone (66). The gentamicin resistance-conferring *aacC1* gene was first replaced with the chloramphenicol resistance-conferring gene *cat* and its native promoter from pTOX5 (67). Next, the constitutive promoter of the *rpsL* gene from *E. hormaechei* strain NR2335 was PCR amplified and ligated into the 5′ end of the multiple cloning site of the vector, thereby generating pUC18R6KS12Cm. The WT *wzy* gene was then ligated into the multiple cloning site of pUC18R6KS12Cm 3′ to the constitutive *rpsL* promoter, thereby generating pUC18R6KS12Cm-*wzy*.

The P_S12_-*wzy* cassette was delivered to the NR3055 genome via electroporation, and this followed a previously established protocol for electrocompetent cell preparation (68). Briefly, 50 mL TSB were inoculated with three NR3055 colonies and grown at 37°C with shaking at 180 rpm for 3 h. Following growth, the cultures were placed on ice for 5 min and centrifuged at 4°C and 4,000 rpm for 15 min. The supernatants were discarded, and the pellets were resuspended in 50 mL ice cold water and then centrifuged again at 4°C and 4,000 rpm for 15 min. Following the final centrifugation, the pellets were resuspended in 500 μL ice cold water. In 2 mm cuvettes, 50 μL of freshly prepared electrocompetent NR3055 cells were mixed with approximately 200 ng pUC18R6KS12Cm-*wzy* and approximately 300 ng of the pTNS2 helper transposase plasmid (69), and cells were electroporated at 25 μF, 200 Ω, and 2.5 kV, and approximately 950 μL SOC broth were added to the cuvettes immediately after electroporation. The cells were transferred to microcentrifuge tubes and grown at 37°C with shaking at 180 rpm for 45 min. Following outgrowth, the cells were plated onto LB agar supplemented with 50 μg/mL chloramphenicol. Clones containing the P_S12_-*wzy* cassette were verified via PCR using primers annealing to the Tn7R end of the transposon and to the flanking *glmS* gene. We named the successfully complemented strain NR3055 *att*Tn7::P_S12_-*wzy* (which can be shortened to NR3055::*wzy*).

### Determining the percent cell sedimentation.

Cultures of NR3055, NR3055 SR2, and NR3055*::wzy* were grown overnight at 37°C and 180 rpm. The next morning, the cultures were mixed via pipetting and diluted 1:10 in PBS in spectrophotometer cuvettes. The OD_600_ values of the mixtures were measured to determine the pre-sedimentation OD_600_ value. To measure the post-sedimentation OD_600_ value, the cultures were incubated statically on the bench for 4 h. Carefully, without disturbing or mixing the cultures, 100 μL from the tops of the cultures were diluted 1:10 in PBS in spectrophotometer cuvettes, and the OD_600_ values were measured. To calculate the percentage of cells in suspension, the 4 h (post-sedimentation) OD_600_ measurements were divided by the pre-sedimentation OD_600_ measurements and multiplied by 100. That value was then subtracted from 100 to determine the percentage of cells that sedimented to the bottom of the tube.

### CPS staining.

Extracellular polysaccharides were extracted using a previously described method (30). Cultures of NR3055, NR3055 SR2, and NR3055::*wzy* were grown overnight at 37°C and 180 rpm. The next morning, the cultures were normalized to an OD_600_ of approximately 5.0. 500 μL of culture were added to 100 μL of 1% zwittergent (pH 2) and incubated at 50°C for 20 min. The samples were centrifuged for 5 min at max speed. 300 μL of supernatant was then transferred to 1.2 mL of absolute ethanol and incubated at 4°C for 20 min to precipitate the polysaccharides. The samples were centrifuged for 10 min at 4°C and 10,000 rpm. The supernatants were decanted, and the pellets were dried for approximately 15 min. The glycoprotein staining portion of this experiment was performed as previously described (70). The pellets were resuspended in 88 μL dH_2_O and were mixed with 20 μL 6× carbohydrate loading dye (0.02% bromophenol blue, 2 M sucrose) and 12 μL 10× Tris-glycine running buffer (Fisher Scientific). 15 μL of each sample were loaded onto NuPage 7% Tris-acetate protein gel (Thermo Fisher Scientific) and run at 120 V for 2 h. The gels were stained using a Pierce Glycoprotein Staining Kit (Thermo Fisher Scientific) per the user instructions.

### Animal housing conditions.

The animals received PicoLab Rodent Diet 20 (LabDiet) and acidified water *ad libitum*. The animals were housed under normal light cycle conditions (12 h on/12 h off) and a temperature of 21°C (± 2°C).

### Murine peritonitis model.

8-week-old female C57BL/6J mice (The Jackson Laboratory) were infected intraperitoneally with 10^8^ CFU (in 300 μL volume) of NR3055, NR3055 SR2, or NR3055*::wzy.* For the survival experiments, the mice were monitored for morbidity and weight loss. Mice that exhibited severe morbidity and/or reached ≤80% of their starting weights were euthanized. Severe morbidity and further euthanasia were determined by hunched posture, lack of movement, ruffled fur, and inability to eat or drink. 2 experiments were conducted with 5 mice infected per strain, resulting in 10 mice total being infected, per strain.

For the experiments to determine bacterial dissemination into tissues, mice were euthanized at 2 hpi and 16 hpi, and blood, peritoneal lavage fluid, spleens, kidneys, livers, lungs, and hearts were collected from all of the mice. Blood samples were incubated on ice in 0.1% saponin for 30 min before plating, and solid tissues were homogenized before plating. Blood, peritoneal lavage fluid, and organ homogenates were serially diluted in PBS and plated on TSA for CFU. As with the survival experiments, 10 total mice were infected per strain for a given time point across two independent experiments.

### C3b deposition quantification via flow cytometry.

NR3055, NR3055 SR2, and NR3055::*wzy* cell suspensions in PBS at an OD_600_ of approximately 0.1 were incubated with 2% C5-depleted serum (Complement Technology, Inc., Tyler, TX), 2% heat-inactivated C5-depleted serum, or no serum for 30 min at 37°C. The bacteria were washed once with RPMI and were then incubated with 3 μg/mL purified FITC anti-complement C3b/iC3b Antibody (BioLegend, San Diego, CA) for 30 min at 4°C while rocking. The fluorescence was analyzed via flow cytometry to measure the binding of antibodies to the C3b bound bacteria.

### Purification of human neutrophils.

LeukoPaks containing peripheral white blood cells from anonymous donors were obtained from the New York Blood Center. LeukoPak samples were gently mixed with an equal volume of 0.9% NaCl with 3% dextran to allow for the sedimentation of the red blood cells. The top fraction of cells containing polymorphonuclear cells (PMNs) and peripheral blood mononuclear cells (PBMCs) were transferred to new 50 mL conical tubes and were washed once with PBS. The cells were resuspended in Hanks’ Balanced Salt Solution (ThermoFisher, Waltham, MA) and were then layered on top of Ficoll (Ficoll-Paque PLUS, GE Healthcare, Chicago, IL). The cells were then centrifuged for 30 min to separate the cell types by density. The supernatant was removed, and the PMN pellet was washed in PBS. The PMNs were then resuspended in Gibco ACK Lysis Buffer (ThermoFisher, Waltham, MA) to remove any remaining red blood cells. Purified PMNs were resuspended in RPMI with 0.1% human serum albumin (HAs, SeraCare Life Technologies, Inc., Milford MA) and 10 mM HEPES buffer.

### Opsonophagocytosis assay.

This assay was carried out as previously described (71). Briefly, GFP-producing NR3055 (NR3055 pJH026) and GFP-producing NR3055 SR2 (NR3055 SR2 pJH026) were opsonized with 2% C5-depleted serum (Complement Technology, Inc., Tyler TX) for 30 min at 37°C. Purified human PMNs were infected with each strain at an MOI of 10. The samples were incubated at 37°C in a roller drum incubator and at 0 hpi, 0.5 hpi, 1 hpi, 2 hpi, and 3 hpi, the fluorescence was analyzed via flow cytometry to detect the bacterial uptake through gating for PMNs by forward versus side scatter. The pJH026 vector (72), which contained *gfp* driven by the P_em7_ promoter, was introduced into NR3055 and NR3055 SR2 via electroporation, as described above.

### C3-deficient murine peritonitis infection.

C3-deficient C57BL/6J breeding pairs were purchased from The Jackson Laboratory (B6.129S4-*C3^tm1Crr^*/J, strain number 029661) and bred in our animal facility. Male 8-week-old, C3-deficient offspring (*n* = 6) were infected intraperitoneally with 2.5 × 10^8^ CFU of NR3055, whereas female 8-week-old, C3-deficient offspring (*n* = 9) were infected intraperitoneally with 10^8^ CFU of NR3055. As a comparison, male (*n* = 5) and female (*n* = 15) WT C57BL/6J mice were infected with the same doses, based on sex. The mice were monitored for weight loss and survival. Mice that exhibited severe morbidity and/or reached ≤80% of their starting weights were euthanized. Each experimental group had either 15 or 20 mice.

### Murine model of recurrent infection.

8-week-old female C57BL/6J mice (The Jackson Laboratory) were infected intraperitoneally with a nonlethal dose (10^6^ CFU in 300 μL volume) of NR3055 or NR3055*::wzy.* For boosting, the same mice were reinfected with 10^6^ CFU (in a 300 μL volume) of NR3055 or NR3055*::wzy* at 3 weeks post primary infection. At 2 weeks post-boost, all of the mice were challenged with a lethal dose (2.5 × 10^8^ CFU in a 300 μL volume) of NR3055::*wzy*. The mice were monitored for morbidity and weight loss. Mice that exhibited severe morbidity, as defined above, and/or reached ≤80% of their starting weights were euthanized. Two experiments were conducted with 5 mice infected per strain, resulting in 10 total mice being infected per strain. A total of 15 mice were infected, reinfected, and challenged with NR3055::*wzy*.

For the passive immunization studies, surviving mice that had been previously exposed to and infected with NR3055::*wzy* were terminally bled via cardiac puncture. Serum was collected and pooled. 8-week-old, female C57BL/6J mice were pretreated intravenously via tail vein injection with 250 μL of either pooled serum from NR3055::*wzy*-infected mice or naive serum that was isolated from uninfected mice. 24 hours posttreatment, the mice were infected with 10^8^ CFU of NR3055::*wzy.* Mice that exhibited severe morbidity, as defined above, and/or reached ≤80% of their starting weights were euthanized. 2 experiments were conducted with 5 mice being infected per treatment group, resulting in 10 mice in total being infected per group.

### Statistics.

GraphPad Prism 9 was used for the statistical analyses. All of the statistical details of the experiments can be found in the figure legends.

### Data availability.

The assemblies and sequencing reads are available in NCBI under BioProject PRJNA867733.
