# Supplementary figures and images for: Capsular Polysaccharide Is Essential for the Virulence of the Antimicrobial-Resistant Pathogen Enterobacter hormaechei
Source: mBio. 2023 Feb 13;14(2):e02590-22. doi: 10.1128/mbio.02590-22 (PMC10127600; doi:10.1128/mbio.02590-22)

**A****NR3055**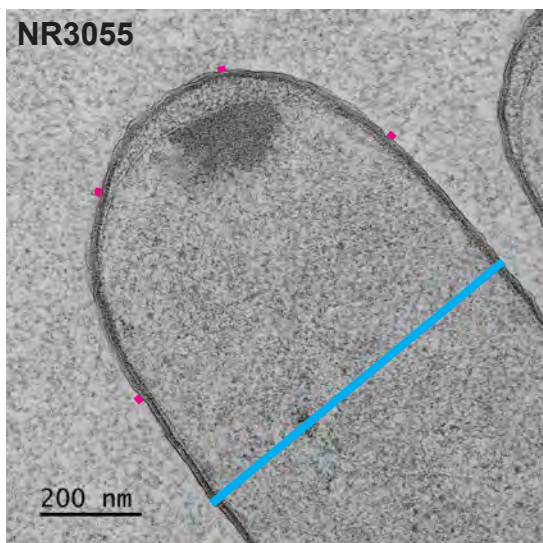**B****NR3055**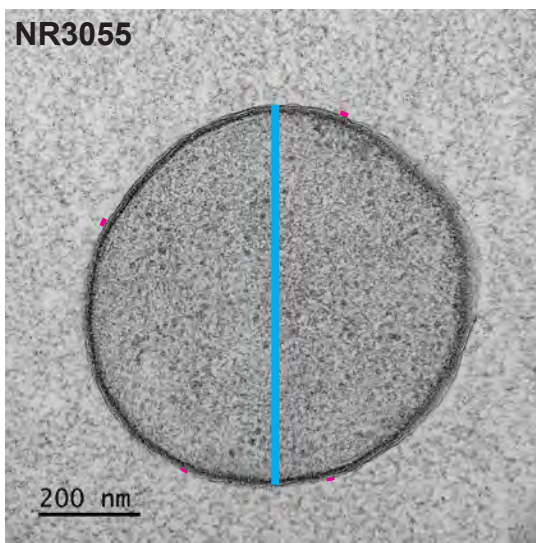**C****NR3055 SR2**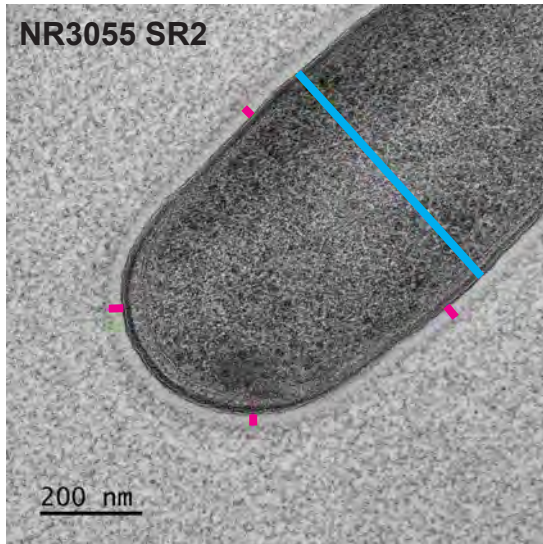**D****NR3055 SR2**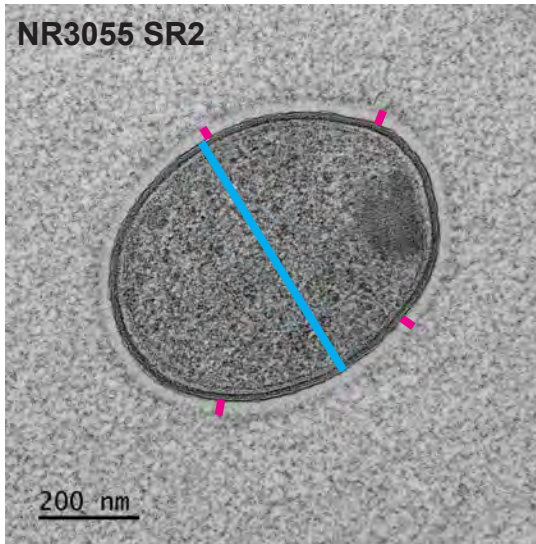

Supplement: FIG S2 [file mbio.02590-22-s0002.pdf]
