# Supplementary material for: Capsular Polysaccharide Is Essential for the Virulence of the Antimicrobial-Resistant Pathogen Enterobacter hormaechei
Source: mBio. 2023 Feb 13;14(2):e02590-22. doi: 10.1128/mbio.02590-22 (PMC10127600; doi:10.1128/mbio.02590-22)
Supplement: FIG S1 [file mbio.02590-22-s0001.pdf]

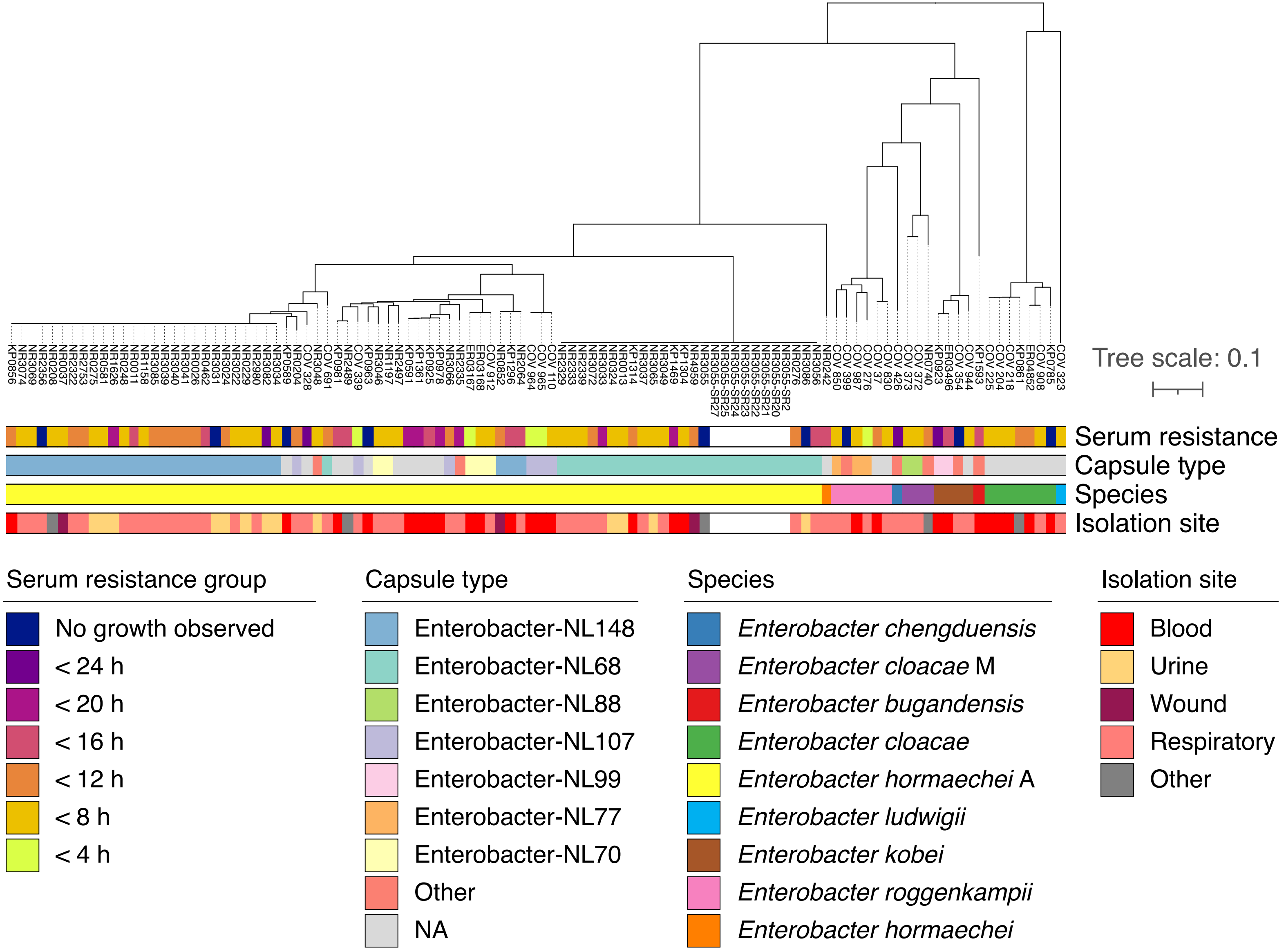
Tree scale: 0.1
Serum resistance
Capsule type
Species
Isolation site
Serum resistance group
No growth observed
< 24 h
< 20 h
< 16 h
< 12 h
< 8 h
< 4 h
Capsule type
Enterobacter-NL148
Enterobacter-NL68
Enterobacter-NL88
Enterobacter-NL107
Enterobacter-NL99
Enterobacter-NL77
Enterobacter-NL70
Other
NA
Species
Enterobacter chengduensis
Enterobacter cloacae M
Enterobacter bugandensis
Enterobacter cloacae
Enterobacter hormaechei A
Enterobacter ludwigii
Enterobacter kobei
Enterobacter roggenkampii
Enterobacter hormaechei
Isolation site
Blood
Urine
Wound
Respiratory
Other
