# Supplementary material for: Capsular Polysaccharide Is Essential for the Virulence of the Antimicrobial-Resistant Pathogen Enterobacter hormaechei
Source: mBio. 2023 Feb 13;14(2):e02590-22. doi: 10.1128/mbio.02590-22 (PMC10127600; doi:10.1128/mbio.02590-22)
Supplement: FIG S3 [file mbio.02590-22-s0003.pdf]

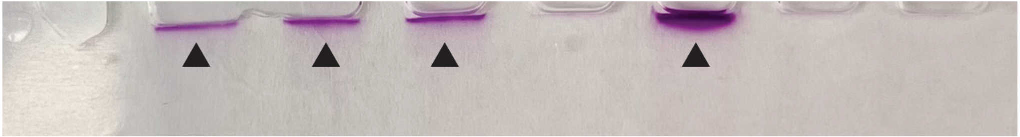

	NR3055	NR3055 SR2	NR3055::wzy	NR3033	NR3082	NR2980	KP1593	KP0589
**Sequence Type**	78	78	78	78	171	171	917	408
**Capsule Type**	NL68	NL68	NL68	NL68	NL148	NL148	NL109	N/A
**Serum Susceptibilty**	S	R	R	R	S	R	S	S
