# Supplementary material for: Capsular Polysaccharide Is Essential for the Virulence of the Antimicrobial-Resistant Pathogen Enterobacter hormaechei
Source: mBio. 2023 Feb 13;14(2):e02590-22. doi: 10.1128/mbio.02590-22 (PMC10127600; doi:10.1128/mbio.02590-22)
Supplement: TABLE S3 [file mbio.02590-22-s0006.docx]

**Table S3. Bacterial strains and plasmids used in this study.**

**Strain Source**

*Enterobacter*

COV_426 This study

COV_399 This study

COV_110 This study

COV_964 This study

COV_965 This study

COV_339 This study

NR3066 [17]

NR0204 [17]

KP1296 [17]

NR0852 [17]

NR2064 [17]

KP0856 [17]

NR0011 [17]

NR0026 [17]

NR3066 [17]

NR0037 [17]

NR0208 [17]

NR0229 [17]

NR0248 [17]

NR0275 [17]

NR0462 [17]

NR0581 [17]

NR1158 [17]

NR1626 [17]

NR2322 [17]

NR2356 [17]

NR2753 [17]

NR2980 [17]

NR3022 [17]

NR3024 [17]

NR3031 [17]

NR3034 [17]

NR3039 [17]

NR3041 [17]

NR3069 [17]

NR3074 [17]

NR3085 [17]

NR3048 [17]

COV_354 This study

KP1304 [17]

KP1314 [17]

KP1469 [17]

NR0276 [17]

NR0324 [17]

NR3037 [17]

NR3049 [17]

NR3056 [17]

NR3065 [17]

NR3086 [17]

NR4959 [17]

COV_691 This study

ER03167 This study

ER03168 This study

NR3046 [17]

NR1197 [17]

COV_912 This study

NR0740 [17]

COV_276 This study

COV_850 This study

COV_987 This study

COV_372 This study

COV_373 This study

ER03496 This study

KP0923 [17]

COV_908 This study

ER04852 This study

KP0981 [17]

NR2489 [17]

KP0925 [17]

NR0242 [17]

KP0785 [17]

COV_204 This study

COV_218 This study

COV_225 This study

KP0861 [17]

KP0591 [17]

KP0978 [17]

KP0963 [17]

COV_328 This study

COV_323 This study

COV_37 This study

COV_830 This study

COV_944 This study

NR2497 [17]

KP1593 [17]

NR3040 [17]

NR3082 [17]

NR0013 [17]

NR2329 [17]

NR2333 [17]

NR2339 [17]

NR3033 [17]

NR3055 [17]

NR3072 [17]

NR2335 [17]

KP1361 [17]

KP0589 [17]

NR3055-SR2 This study

NR3055-SR20 This study

NR3055-SR21 This study

NR3055-SR22 This study

NR3055-SR23 This study

NR3055-SR24 This study

NR3055-SR25 This study

NR3055-SR27 This study

*Escherichia coli*

DH5α λpir Lab stock

**Plasmid Source**

pUC18R6K-mini-Tn7T-Gm [67]

pTNS2 [70]

pTOX5 [68]

pUC18R6K-mini-Tn7T-Cm This study

pUC18R6KS12Cm This study

pUC18R6KS12Cm-*wzy* This study

pJH026 [73]
